# The impact of cytoreductive nephrectomy on survival outcomes in patients with metastatic renal cell carcinoma receiving immunotherapy: An evidence-based analysis of comparative outcomes

**DOI:** 10.3389/fimmu.2023.1132466

**Published:** 2023-03-14

**Authors:** Kun-peng Li, Si-yu Chen, Chen-yang Wang, Xiao-ran Li, Li Yang

**Affiliations:** Department of Urology, The Second Hospital of Lanzhou University, Lanzhou, China

**Keywords:** metastatic renal cell carcinoma, cytoreductive nephrectomy, immunotherapy, overall survival, outcomes

## Abstract

**Purpose:**

The prognostic impact of cytoreductive nephrectomy (CN) for metastatic renal cell carcinoma (mRCC) in the era of immunotherapy is yet to be determined. The aim of our study is to evaluate the correlation between CN and outcomes in the setting of mRCC treated with immunotherapy.

**Methods:**

We conducted a systematic search of the Science, PubMed, Web of Science, and Cochrane Library databases to identify relevant studies published in English up to December 2022. The results were presented as hazard ratio (HR) with 95% confidence intervals (CIs) for overall survival (OS) was extracted to assess their relevance. The study was registered with PROSPERO (CRD42022383026).

**Results:**

A total of 2397 patients were included in eight studies. The CN group was observed to be correlated with superior OS compared to the No CN group (HR = 0.53, 95% CI 0.39–0.71, p < 0.0001). Subgroup analysis according to the type of immunotherapy, sample size, and treatment line of immune checkpoint inhibitor revealed that CN group had a superior OS in all subgroups.

**Conclusion:**

CN is associated with a better outcome in terms of OS benefit in selected patients with mRCC treated by immunotherapy, but further studies are required to verify the conclusions.

**Systematic review registration:**

https://www.crd.york.ac.uk/prospero/, identifier CRD42022383026.

## 1 Introduction

In the early twentieth century, the immunogenicity of renal cell carcinoma was discovered, leading to the establishment of interferon-alfa (IFN-alfa) and interleu-kin-2 as first-line therapies for metastatic renal cell carcinoma (mRCC) (1–3). However, the role of cytoreductive nephrectomy (CN) in the treatment of mRCC remains controversial. Two randomized clinical trials (RCTs) conducted by Mickish et al. (4) and Flanigan et al. (5) demonstrated that the combination of CN and IFN-alfa significantly improved overall survival (OS) of patients with mRCC compared to IFN-alfa therapy alone.

Over the past decade, the treatment paradigm for mRCC has significantly evolved, with targeted therapy becoming the new standard of care (6). The introduction of more effective targeted therapies has called into question the role of CN in this context. More recently, the results from a prospective RCT, CARMENA (7), demonstrated that patients with mRCC who received targeted therapy alone had comparable OS to those who received CN followed by targeted therapy. Additionally, another RCT, SURTIME (8), also questioned the value and the optimal timing of CN in relation to the initiation of systemic therapy. However, the universality and availability of both trials have been questioned due to delayed recruitment and unbalanced proportion of patients with poor-risk diseases in CARMENA study population (9). Méjean et al. (10) conducted a study which stratified patients according to the International Metastatic Renal Cell Carcinoma Database Consortium (IMDC), demonstrating that some patients could still benefit from CN. Additionally, a meta-analysis encompassing 14 studies showed that CN can be beneficial for patients receiving targeted therapy (11). More recently, immune checkpoint inhibitors (ICIs) have revolutionized the treatment of mRCC. The outcomes from the CheckMate-025 study have led to the approval of nivolumab as the first ICIs for mRCC patients (12, 13). ICIs therapy, either alone or in combination with targeted therapy, has demonstrated superior efficacy and has been used as a first-line treatment for mRCC (14, 15). Despite the potential benefits of combined therapy of CN and immunotherapy, the impact of CN on patient outcomes remains controversial. Moreover, the small sample sizes of different clinical centers limit the reliability of any conclusions drawn.

This systematic review and meta-analysis aim to integrate the data from comparative studies to evaluate the relationship between CN and outcomes in the setting of mRCC treated with immunotherapy, thereby providing latest evidence for clinical decision-making.

## 2 Methods

The present study was conducted in accordance with the Preferred Reporting Items for Systematic Reviews and Meta-Analyses (PRISMA) statement 2020 (16, 17), and was registered in PROSPERO (ID: CRD42022383026).

### 2.1 Literature search strategy, study selection and data collection

We systematically searched the databases such as Science, PubMed, Web of Science, and Cochrane Library to identify published studies till December 2022. The search terms were as follows: ((Renal cell carcinoma OR kidney carcinoma OR renal cell cancer) AND (Metastasis OR advanced) AND (Cytoreductive nephrectomy OR nephrectomy OR radical nephrectomy) AND (Immune checkpoint inhibitor OR immunotherapy OR immune-oncology OR PD-1 inhibitor OR PD-L1 inhibitors OR anti-PD-1 inhibitor)). Furthermore, we manually searched the relevant references and abstracts to avoid any omissions and expand the search scope.

We used the PICOS approach to define the inclusion criteria. P (patients): All the patients were diagnosed with mRCC; I (intervention): patients were undergone CN, either prior to (upfront) or following the initiation of immunotherapy (deferred). The immunotherapy was defined as cytokine-based therapy (IFN-alfa and interleu-kin-2), and ICIs; C (comparator): immunotherapy without CN; O (outcome): survival outcomes; S (study type): randomized controlled trials (RCTs), prospective studies and retrospective studies. Exclusion criteria include (1) duplicate studies and non-comparative studies, (2) the type of letters, comments, meeting abstracts, case reports and reviews, and (3) studies without detailed data for analysis.

Two evaluators (K.L. and S.C.) independently extracted the data from each qualified publication. The following data were extracted: (1) first author, year of publication, center, country, and study period. (2) age, sample size, gender, and follow-up period. (3) IMDC risk score, metastatic sites, number of sites of metastasis, and type of immunotherapy. (4) overall survival (OS). Any discrepancies and disagreements were resolved by discussion with a third evaluators (Y.L.).

In these studies, the risk of bias in non-randomized studies of interventions (ROBINS-I) was used to evaluate the quality of the non-RCTs (18). Furthermore, the Cochrane Collaboration tool was used to evaluate the quality of the RCTs (19). Two independent reviewers access the quality of included literatures, and any discrepancies were settled through discussion.

In the present study, the statistical analysis was processed using Cochrane Collaborative RevMan5.4 software. The hazard ratio (HR) was calculated for all the survival outcomes, and the results were presented with 95% confidence intervals (CIs). Considering the predictable significance between-trial heterogeneity, we used the random-effects model in all analyses. The I^2^ test was used to evaluate the heterogeneity of each indicator among the studies (20), and statistical significance was considered p < 0.05. Publication bias was evaluated using the Begg’s method funnel plot.

### 2.2 Subgroup analysis

The subgroup analysis was performed according to the type of immunotherapy, sample size, and treatment line of ICIs.

## 3 Results

### 3.1 Baseline characteristics

**Figure 1:**
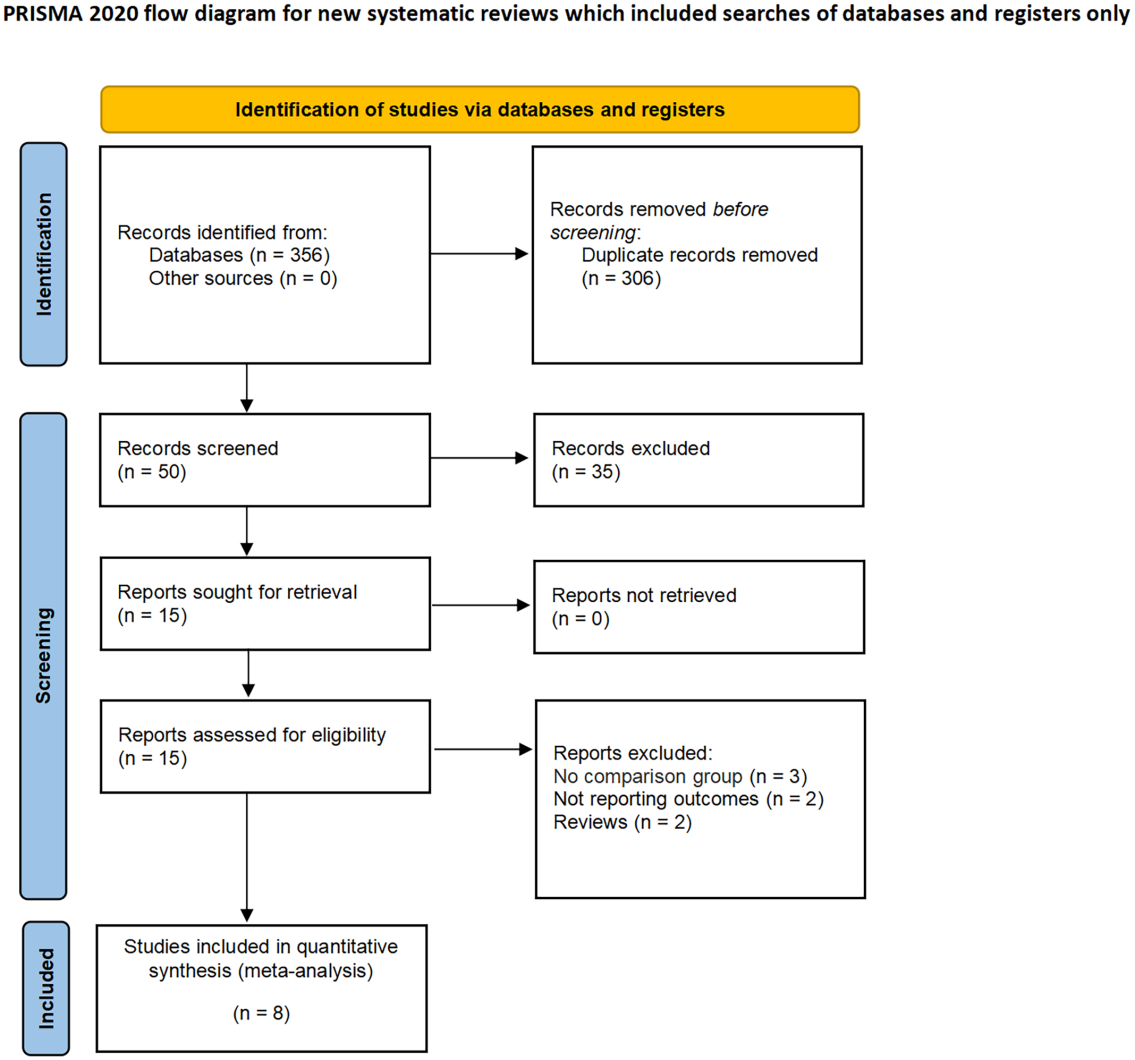
PRISMA flow diagram for the systematic review.

A total of 356 studies were initially identified through electronic search, with 15 remaining after removal of duplicates. After having read and screen the abstracts and full text, eight studies (two RCTs and six non-RCTs) involving 2397 patients were included in the meta-analysis (1606 CN vs. 791 No CN) (**Figure 1**) (4, 5, 21–26). Six non-RCTs were retrospective comparisons. All the studies were from multi-institutional, with six using ICIs as immunotherapy and the others using IFN-alfa (4, 5). The present studies were conducted in different countries, including the USA, Italy, Japan, and Netherlands, with a follow-up period ranging from 12 to 40 months. **Table 1** summarizes the key characteristics of included studies, including their preoperative variables (country, age, sample size, and gender). **Table 2** summarize the oncologic outcomes and interventions (IMDC risk score, metastatic sites, number of sites of metastasis, and type of immunotherapy). In the studies included, three studies compared the outcomes of deferred versus upfront CN in patients. **Tables S1** summarize the demographic characteristics and oncologic outcomes of the deferred and upfront CN groups (age, gender, race, IMDC risk score, clear cell, metastatic sites, number of sites of metastasis, time from diagnosis to systemic therapy, follow-up duration).

**Table 1:** The trials included in the systemic review.

Reference	Year	Country	Center	Study Period	Patients		Age (y)		Male/Female		Clear cell (n)		Follow-up duration	
					CN	No CN	CN	No CN	CN	No CN	CN	No CN	CN	No CN
Gross	2022	USA	multi-institutional	2000 to 2020	232	135	Median (IQR): 60(52-66)	Median (IQR): 62(56-68)	180/52	107/28	159	97	39.9(18.2-66.9) months	13.1(6.8-20.4) months
Bakouny	2022	USA	multi-institutional	January 2009 to January 2020	234	203	Median (IQR): 60(53-66)	Median (IQR): 63(56-70)	172/61	146/57	204	110	Mean: 12 months	
Rebuzzi	2022	Italy	multi-institutional	cut-off (July 2020)	490	66	Median (range): 62(18-85)	Median (range): 66(40-84)	347/143	44/22	407	57	Mean: 16.3 months	
Yoshino	2022	Japan	multi-institutional	September 2016 to July 2021	21	13	Median (IQR): 64(53.5-69.5)	Median (IQR): 70(56.5-73)	13/8	8/5	18	8	Mean: 12.0 months	
Stellato	2021	Italy	multi-institutional	February 2017 to January 2020	246	41	Median: 69.4		206/81		246		Mean: 24.7 months	
Singla	2020	USA	multi-institutional	2015 to 2016	221	170	Median (IQR): 57(51-64)	Median (IQR): 64(57-72)	167/54	120/50	NA		Mean: 14.7 months	
Mickisch	2001	Netherlands	multi-institutional	June 1995 to July 1998	42	42	Median (range): 61(36-76)	Median (range): 56(29-74)	33/9	27/15	NA		followed up until death	
Flanigan	2001	USA	multi-institutional	June 1991 to October 1998	120	121	Mean(range): 58.8(37-80)	Mean(range)59: (29-87)	83/37	84/37	NA		Mean: 368 days	

CN, cytoreductive nephrectomy; IQR, interquartile range.

**Table 2:** The trials included in the systemic review.

Reference	IMDC		Metastatic sites		Number of sites of metastasis		Type of immunotherapy	
	CN	No CN	CN	No CN	CN	No CN	CN	No CN
Gross	Favorable: 13; Intermediate: 178; Poor: 38; Missing: 3	Favorable: 4; Intermediate: 89; Poor: 40; Missing: 2	Lymph node: 60; Lung: 150; Liver: 22; Bone: 72; CNS: 5; Muscle: 6; Other kidney: 1; Others: 57	Lymph node: 41; Lung: 82; Liver: 25; Bone: 53; CNS: 12; Muscle: 2; Other kidney: 3; Others: 36	One: 115; 2 or more: 103; Unknown: 14	One: 47; 2 or more: 82; Unknown: 6	Ipilimumab and Nivolumab, Nivolumab; First-line: 47; Second line: 34; Third line or later: 151	Ipilimumab and Nivolumab, Nivolumab; First-line: 56; Second line: 30; Third line or later: 49
Bakouny	Favorable: 18; Intermediate: 143; Poor: 39; Missing: 34	Favorable: 1; Intermediate: 78; Poor: 88; Missing: 36	bone, brain, or liver metastases: 87	bone, brain, or liver metastases: 120	One: 52; 2 or more: 171; Unknown: 11	One: 39; 2 or more: 155; Unknown: 9	combination of nivolumab and ipilimumab as first-line therapy	
Rebuzzi	Favorable: 127; Intermediate: 312; Poor: 51	Favorable: 2; Intermediate: 46; Poor: 18	bone metastases: 331	bone metastases: 30	NA		Nivolumab; Second line: 333; Third line: 106; further line: 51	Nivolumab; Second line: 51; Third line: 12; further line: 3
Yoshino	Intermediate: 14; Poor: 7	Intermediate: 4; Poor: 9	Liver: 3; Bone: 7;	Liver: 1; Bone: 0;	One: 8; 2 or more: 13; Unknown: 11	One: 3; 2 or more: 10; Unknown: 9	combination of nivolumab and ipilimumab as first-line therapy	
Stellato	Favorable: 82; Intermediate: 176; Poor: 29		Lymph node: 128; Lung: 122; Liver: 33; Bone: 84; brain: 12; gland: 37; peritoneum: 14		NA		Ipilimumab and Nivolumab, Nivolumab; Second line: 195; Third line: 73; further line: 19	
Singla	NA		Lung: 147; Liver: 17; Bone: 73; brain: 14	Lung: 100; Liver: 34; Bone: 17; brain: 62	One: 131; 2 or more: 55; Unknown: 35	One: 82; 2 or more: 58; Unknown: 30	Ipilimumab and Nivolumab	
Mickisch	NA		Lymph node: 11; Lung: 33; Liver: 5; Other abdominal: 4; Skin: 2; Bone: 9; Central nervous system: 0	Lymph node: 18; Lung: 34; Liver: 4; Other abdominal: 5; Skin: 2; Bone: 10; Central nervous system: 1	NA		interferon alfa	
Flanigan	NA		Only lung metastases:79	Only lung metastases: 81	NA		interferon alfa	

CN, cytoreductive nephrectomy; IMDC, International Metastatic Renal Cell Carcinoma Database Consortium.

No significant difference was found in age (p = 0.05), clear cell (p = 0.12), bone metastasis (p = 0.58), and lung metastasis (p = 0.21). However, the liver metastasis was significantly less in the CN group compared to the No CN group (p = 0.02) (**Table 3**).

**Table 3:** Comparison of baseline patient.

Baseline characteristic	CN VS NO CN	Heterogeneity I^2^ (%)	*p* value
Age WMD (95% CI)	-2.50(-4.94 to -0.05)	80	0.05
Clear cell OR (95% CI)	1.79(0.86 to 3.74)	71	0.12
Metastatic sites: bone (95% CI)	1.19(0.64 to 2.24)	78	0.58
Metastatic sites: lung (95% CI)	1.18(0.91 to 1.52)	0	0.21
Metastatic sites: liver (95% CI)	0.51(0.28 to 0.90)	35	0.02

CN, cytoreductive nephrectomy.

### 3.2 Assessment of quality

**Figure 2:**
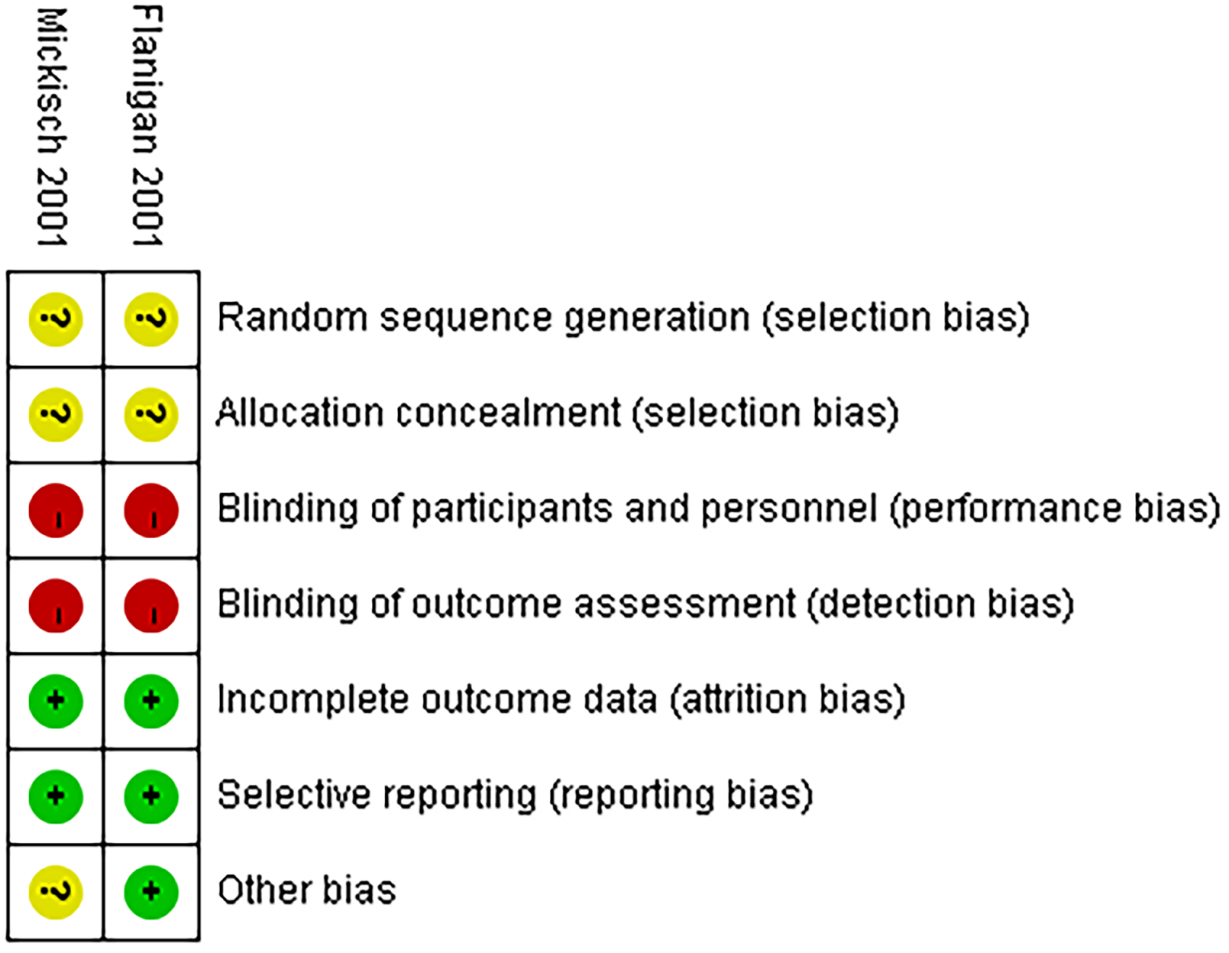
Risk of bias assessment (RCTs).

A comparative analysis was performed on all the non-RCTs, of which five studies had a moderate risk of bias (22–26) and one study had a low risk of bias (**Table S2**). All the non-RCTs were published between 2020 and 2022. Additionally, the two RCTs were not double-blinded, which increased the bias risk, thereby classifying them as high risk (**Figure 2**) (4, 5).

### 3.3 Outcome analysis

#### 3.3.1 Overall survival

**Figure 3:**
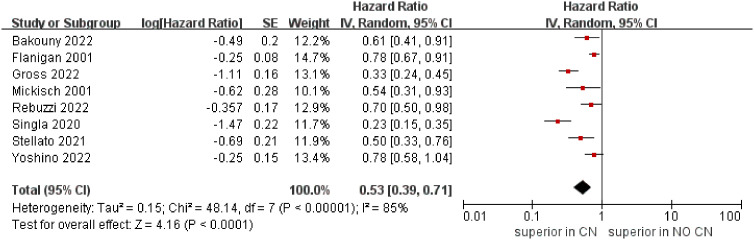
Forest plots of overall survival.

The meta‐analysis included eight studies that reported the OS (4, 5, 21–26). The combined results demonstrated that the CN group was associated with superior OS compared to the No CN group (HR = 0.53, 95% CI 0.39–0.71, p < 0.0001), and with high heterogeneity (I^2^ = 85%) (**Figure 3**).

#### 3.3.2 Subgroup analyses

**Figure 4:**
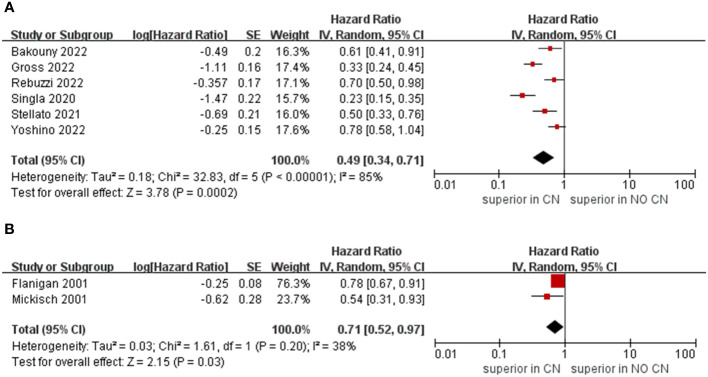
Forest plots of overall survival in subgroup analysis. **(A)** ICIs, **(B)** IFN-alfa.

**Figure 5:**
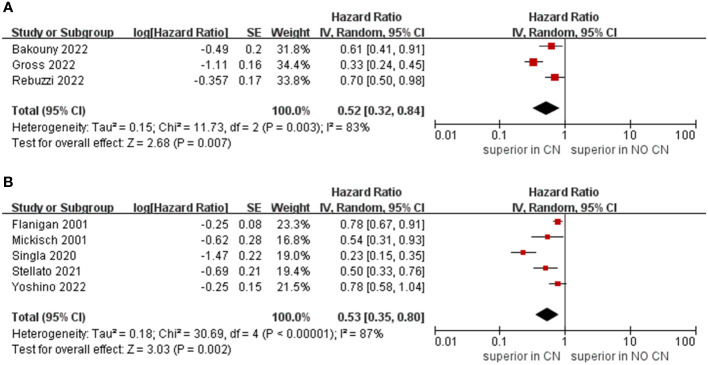
Forest plots of overall survival in subgroup analysis. **(A)** sample size > 400, **(B)** sample size ≤ 400.

**Figure 6:**
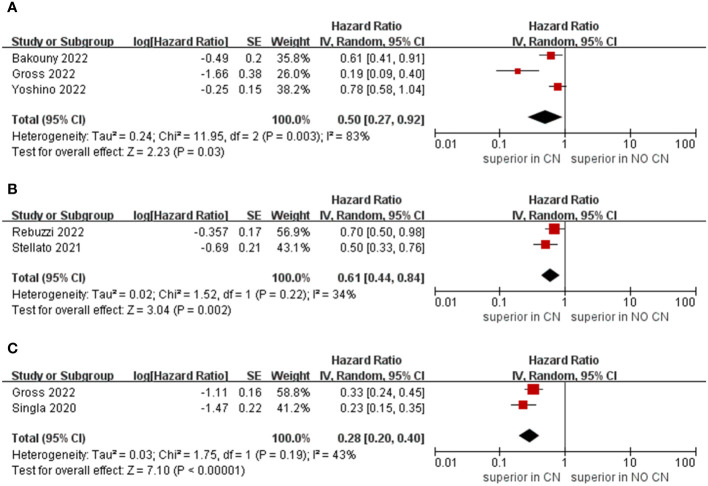
Forest plots of overall survival in subgroup analysis. **(A)** ICIs as first line therapy, **(B)** second and third line therapy, **(C)** first, second and third line or later therapy).

Owing to the insufficient literature included in the meta-analysis, we only preformed subgroup analysis of OS with respect to type of immunotherapy, sample size, and treatment line of ICIs. For studies that include the ICIs, the CN group had significantly lower risk of death compared to the No CN group (HR = 0.49, 95% CI 0.34–0.71, p = 0.0002, I^2^ = 85%) (21–26). In IFN-alfa subgroup, the CN group was also observed to be correlated with superior OS than for the No CN group (HR = 0.71, 95% CI 0.52–0.97, p = 0.03, I^2^ = 38%) (**Figure 4**) (4, 5). In the sample size > 400 subgroup, the CN group had significantly lower risk of death compared to the No CN group (HR = 0.52, 95% CI 0.32–0.84, p = 0.007, I^2^ = 83%) (21–23). Additionally, for the subgroup with a sample size ≤ 400, the CN group was correlated with better OS than for the No CN group (HR = 0.53, 95% CI 0.35–0.80, p = 0.002, I^2^ = 87%) (**Figure 5**) (4, 5, 24–26). In the subgroup analysis of the ICIs as first-line therapy, the CN group had significantly lower risk of death compared to the No CN group (HR = 0.50, 95% CI 0.27–0.92, p = 0.03, I^2^ = 83%) (21, 22, 24). Similarly, the subgroup analysis revealed that both ICIs as second, third line therapy and first, second and third therapy were associated with superior OS compared to the No CN group (HR = 0.61, 95% CI 0.44–0.84, p = 0.002, I^2^ = 34%; HR = 0.28, 95% CI 0.20–0.40, p < 0.00001, I^2^ = 43%) (**Figure 6**) (21, 23) (25, 26).

### 3.4 Sensitivity analysis

We conducted leave-one-out tests to identify the source of heterogeneity and to evaluate the robustness of the results. Ultimately, no substantial change in heterogeneity and pooled HR was found among the studies, regardless of which study was excluded, implying that the source of heterogeneity and the outcomes were stable and reliable. The heterogeneity observed in the outcomes of the studies could be attributed to a variety of factors, including follow-up period, IMDC risk score, metastatic sites, number of sites of metastasis, and type of immunotherapy. Additionally, caution should be taken when interpreting the results of the analyses, as the I^2^ statistic has been observed to be substantially biased in studies with small sample sizes (27).

### 3.5 Publication bias

**Figure 7:**
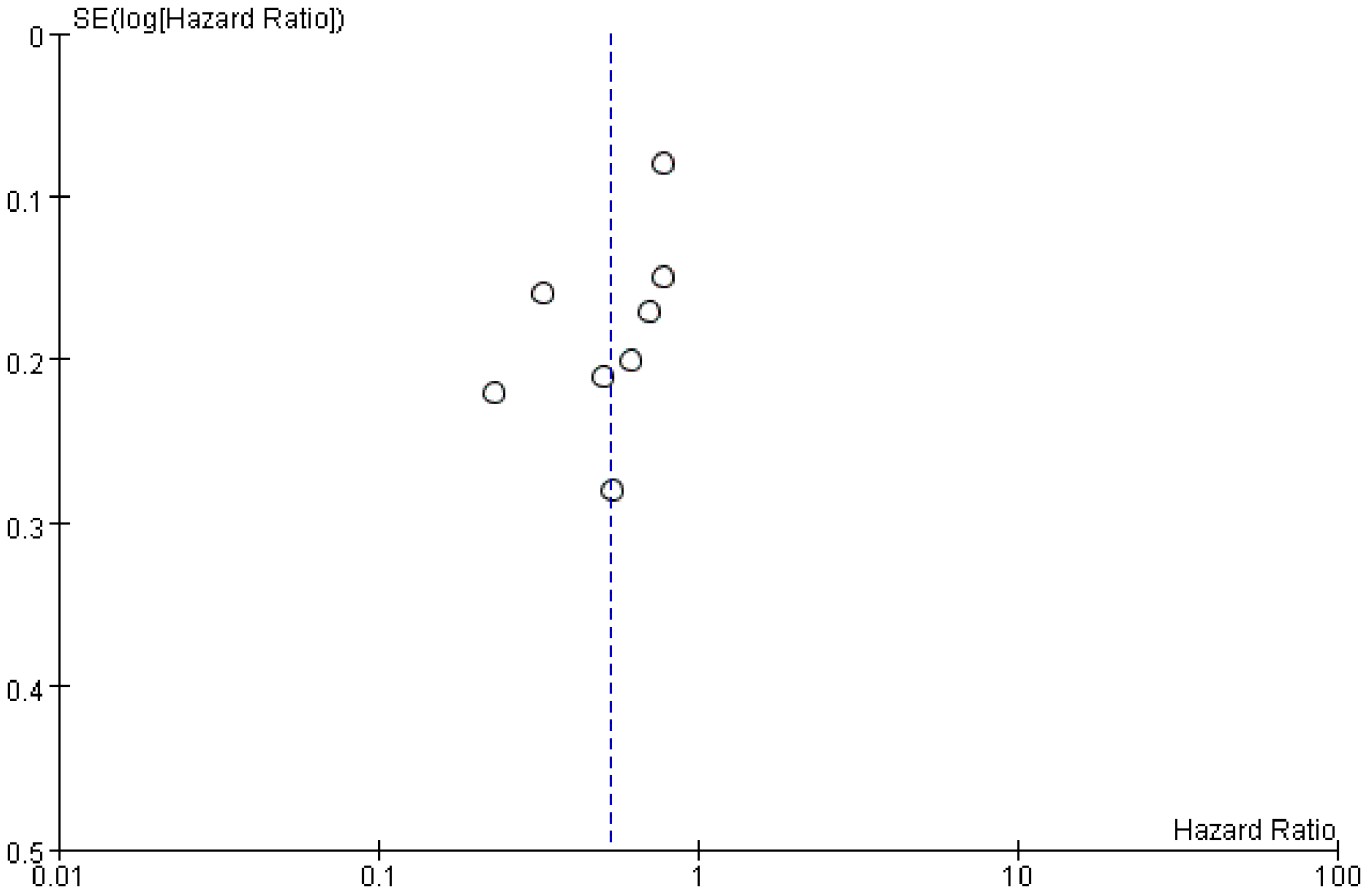
Funnel plot of the studies represented in the meta-analysis.

We examined publication bias by the funnel plot. The findings revealed that the distribution of included studies was almost tapered, but there is still some publication bias (**Figure 7**).

## 4 Discussion

This is the first systematic review and meta-analysis to evaluate the prognostic impact of CN for mRCC in the era of immunotherapy. Furthermore, some significant findings from this analysis need further discussion.

In the early twentieth century, the cytokine-based therapy was the standard of care for mRCC, whereas surgical management was still the treatment option for patients with mRCC. As more effective targeted therapy for mRCC have been developed, however, the role of CN has been called into question. There are still some inconsistent statements about the benefits of CN for patients with mRCC in the targeted therapy era. The CARMENA trial (7) showed that patients who received CN did not get adequate benefit compared to those who received targeted therapy alone. It is worth mentioning that the proportion of patients with poor-risk disease is higher in the CARMENA study population. Moreover, the patients included in CARMENA trial received therapy immediately after CN. Nevertheless, Roussel et al. (28) demonstrated that patients receive CN after upfront systemic therapy may get better survival outcomes. Recently, Janisch et al. (29) reported that treatment with CN and tyrosine kinase inhibitors was associated with superior survival compared to those without CN for specific patient. Ghatalia et al. (30) conducted a retrospective study to evaluate the role of CN in patients with mRCC, including those receiving ICIs and targeted therapy, and demonstrated that CN had a beneficial effect on select patients with mRCC. Taken together, accumulating evidence suggests that the combination of CN and systemic therapy may provide better outcomes in mRCC.

Although CN has been the important treatment for mRCC, the underlying mechanism of its survival benefits remains unknown. Over the years, various hypotheses have been put forward. First, the immune hypothesis was proposed in the 1990s (31), which was supported by Fujikawa et al.’s (32) research results in that patients who did not receive CN had lower levels of response for interleukin-2 than those who did. This was further corroborated by two RCTs (4, 5), and our meta-analysis also demonstrated that CN group was associated with superior OS than the No CN group in IFN-alfa subgroup. Second, primary tumors are associated with promoting inflammation and suppressing the release of cytokines from T cells, which could impede the systemic anti-tumor immune response (33, 34). Marcus et al. (31) conducted a case report to show that spontaneous regression of mRCC lesions upon CN, which further demonstrated the outcomes. Hence, resection of the primary tumor may enhance the immune response of mRCC. Third, the straightforward explanation is that CN can reduce the overall tumor burden and thus extend the duration of time before tumors reach lethal levels (35). Additionally, the efficacy of CN combined with immunotherapy has been verified in other types of metastatic tumors, such as lung cancer and melanoma, providing further evidence for its application in mRCC (36, 37).

The optimal timing of CN in relation to the initiation of systemic therapy is a critical factor that may influence outcomes. Bhindi et al. (38) conducted a study using real-world data and concluded that deferred CN could significantly improve OS compared to upfront CN. However, Bruijn et al. (39) conducted a comparative study to assess the outcomes of patients receiving targeted therapy followed by CN (deferred CN) against those receiving CN followed by targeted therapy (upfront CN), and the results showed that there was no significant difference in OS between the two groups. In the studies included, three studies compared the outcomes of deferred versus upfront CN in selected patients. Two studies reported that deferred CN did not lead to a superior OS than upfront CN in patients (21, 26), while one study suggested that OS rate tended to be higher with deferred CN in comparison to upfront CN (24). Ghatalia et al. (30) also demonstrated that no statistically significant difference in OS was observed between the upfront and deferred groups. Nevertheless, the insufficient literature barred us from conducting analysis to compare outcomes between the two approaches. Furthermore, the small sample size of the included studies renders it difficult to draw a reliable conclusion. The SURTIME trial revealed that deferred CN did not improve 28-week progression-free rate, while the deferred CN could be associated with improved OS compared to immediate CN (8). Ghanem et al. (40) reported that immediate CN resulted in a lower rate of successful systemic therapy and disease control compared to deferred CN. Due to the dearth of existing research, it is not possible to draw reliable conclusions as to which of the two methods could bring OS advantage for patients with mRCC. Therefore, further research is needed to verify the efficacy of each approach.

Patient selection is also a crucial consideration when evaluating the benefits of CN (41). As an invasive procedure for patients with high disease burden, CN carries a higher mortality risk than standard nephrectomy for T1 or T2 renal tumors (42). Furthermore, the survival benefit for some patients with poor risk score is marginal, and CN might bring postoperative complications that could negatively affect quality of life, prompting further scrutiny of its role (43). Therefore, patient selection for CN may have potential bias. In the included studies, four studies utilized the IMDC risk score to access the baseline risk score of the two groups. However, only two studies revealed that the rates of poor IMDC risk score in the CN group was lower than that of the No CN group. Furthermore, although liver metastases were found to be significantly less in the CN group compared to the non-CN group, no significant difference was found in bone metastasis and lung metastasis. Bakouny et al. (22) proposed that patients without adverse (bone, liver or lung) metastases, favorable IMDC risk score, and good physical condition may gain adequate benefit from CN. Going forward, newer scales should be created during the ICIs and targeted therapy era to evaluate which patient may benefit from CN. Additionally, we also need more studies to assess the outcomes.

Recently, ICIs therapy has shown superior efficacy and has been adopted as front-line therapy for mRCC. Cytokine-based therapy has gradually given way to ICIs therapy. In our meta-analysis, we have included the latest evidence on CN for mRCC in the era of ICIs therapy. Moreover, some ongoing studies should also be taken into account. The PROBE trial (NCT04510597) is recruiting patients with intermediate or poor risk according to the IMDC risk score who are receiving deferred CN following the combination of nivolumab and ipilimumab, and compared to the No CN group. The SWOG-1931 trial (NCT04510597) is assessing the impact of CN on patients receiving the combination therapy with avelumab and axitinib, or pembrolizumab and axitinib. Further research is needed to confirm these findings with larger sample sizes and higher-quality studies.

However, the limitations of this study should be noted. First, all the included studies were from large centers and the patients enrolled were not necessarily representative of the general population. Second, most the studies included in the analysis were non-RCTs, which undoubtedly had potential distribution and blindness bias. Third, the lack of data in the studies did not allow for a pooled analysis to compare other survival outcomes, such as progression free survival. Fourth, significant differences were observed between the CN and No CN groups in terms of the prevalence of liver metastases. Additionally, two studies have demonstrated that rates of poor IMDC risk score in the CN group were lower than those of the No CN group, resulting in certain heterogeneity. Lastly, due to the limited literature available, a subgroup analysis regarding the timing of CN relative to immunotherapy could not be conducted, which may lead to subtle differences.

## 5 Conclusions

The combination of CN and immunotherapy for mRCC is associated with a better outcome in terms of OS benefit in selected patients compared to immunotherapy alone. Nevertheless, further research is needed to verify these conclusions, such as larger sample sizes, increased follow-up periods and RCTs.

## Data availability statement

The original contributions presented in the study are included in the article/**Supplementary Material**. Further inquiries can be directed to the corresponding authors.

## Author contributions

K-PL: Protocol development, data collection and management, data analysis and manuscript writing. S-YC: Protocol development, data collection and management, data analysis and manuscript writing. C-YW: Protocol development, data management, data analysis and manuscript writing. X-RL: Data management, data analysis and manuscript writing. LY: Data management, data analysis and manuscript writing. All authors contributed to the article and approved the submitted version.
