# Supplementary material for: The impact of cytoreductive nephrectomy on survival outcomes in patients with metastatic renal cell carcinoma receiving immunotherapy: An evidence-based analysis of comparative outcomes
Source: Front Immunol. 2023 Mar 14;14:1132466. doi: 10.3389/fimmu.2023.1132466 (PMC10043247; doi:10.3389/fimmu.2023.1132466)
Supplement: Supplementary file 1 [file Table_1.docx]

| **Table S1 The baseline characteristics of the CN group were compared between patients who received uCN and those who received dCN** | | | | | | |  |
| --- | --- | --- | --- | --- | --- | --- | --- |
|  |  |  |  |  |  |  |  |
| Reference | Gross | | Yoshino | | Singla | |  |
|  | uCN | dCN | uCN | dCN | uCN | dCN |  |
| Patients | 202 | 30 | 21 | 7 | 197 | 24 |  |
| Age (y) | NA | | 64.0 (53.5-69.5 | 56.0 (47.0-64.0 | 56 (51-63) | 65 (56-70) |  |
| Male/Female | 155/47 | 25/5 | 13/8 | 6/1 | 150/47 | 17/7 |  |
| Race | White:174; Black: 8; Asian: 5; Other: 14; Unknown/Missing: 1 | White:24; Black: 2; Asian: 1; Other: 2; Unknown/Missing: 1 | NA | | White:169; Black: 7; Hispanic: 13; Asian/Other: 8 | White:19; Black: 3; Hispanic: 2; Asian/Other: 0 |  |
| IMDC | Favorable: 12; Intermediate:152; Poor: 35; Unknown: 3 | Favorable: 1; Intermediate:26; Poor: 3; Unknown: 0 | Intermediate: 14; Poor: 7; Unknown: 0 | Intermediate: 1; Poor: 5; Unknown: 1 | NA | |  |
| Clear cell (n) | 134 | 25 | 18 | 5 | NA | |  |
| Metastatic sites: bone | 57 | 15 | 7 | 1 | 56 | 17 |  |
| Metastatic sites: lung | 130 | 20 | NA | | 135 | 12 |  |
| Metastatic sites: liver | 18 | 4 | 3 | 1 | 15 | 2 |  |
| Number of sites of metastasis | One: 101; Two or more: 88; Unknown: 13 | One: 14; Two or more: 15; Unknown: 1 | Two or more: 13 | Two or more: 5 | Two or more: 47 | Two or more: 8 |  |
| Time | Time from  diagnosis to  nephrectomy, months: 0.8 (0.2-1.4) | Time from  diagnosis to  nephrectomy, months:   9.8 (5.1-22.5) | Time from diagnosis to systemic therapy, days: 40 (24-63) | Time from diagnosis to systemic therapy, days: 15 (8-22) | Time from  diagnosis to  nephrectomy, days: 21 (7-38) | Time from  diagnosis to  nephrectomy, days: 123 (107-176) |  |
| Follow-up duration (months) | 39.8 (17.1-66.6) | 41.0 (24.7-73.3) | 12 (5-25) | 19 (18-20) | Mean: 14.7 | |  |
| uCN = upfront cytoreductive nephrectomy; dCN: deferred cytoreductive nephrectomy; IMDC = International Metastatic Renal Cell Carcinoma Database Consortium; Median (interquartile range) | | | | | | |  |
