# Supplementary material for: The impact of cytoreductive nephrectomy on survival outcomes in patients with metastatic renal cell carcinoma receiving immunotherapy: An evidence-based analysis of comparative outcomes
Source: Front Immunol. 2023 Mar 14;14:1132466. doi: 10.3389/fimmu.2023.1132466 (PMC10043247; doi:10.3389/fimmu.2023.1132466)
Supplement: Supplementary file 2 [file Table_2.docx]

| **Table S2 The risk of bias (Non-RCTs)-ROBINS-I** | | | | | | |  |
| --- | --- | --- | --- | --- | --- | --- | --- |
| Bias domain | Gross | Bakouny | Rebuzzi | Yoshino | Stellato | Singla |  |
|  |  |  |  |  |  |  |  |
| Bias due to confounding | Moderate | Moderate | Moderate | Moderate | Moderate | Moderate |  |
|  |  |  |  |  |  |  |  |
| Bias in selection of participants into the study | Low | Low | Low | Low | Low | Low |  |
|  |  |  |  |  |  |  |  |
| Bias in classification of interventions | Low | Low | Low | Low | Low | Low |  |
|  |  |  |  |  |  |  |  |
| Bias due to deviations from intended interventions | Low | Low | Moderate | Moderate | Moderate | Low |  |
|  |  |  |  |  |  |  |  |
| Bias due to missing data | Moderate | Moderate | Low | Moderate | Low | Moderate |  |
|  |  |  |  |  |  |  |  |
| Bias in measurement of outcomes | Low | Low | Moderate | Moderate | Low | Moderate |  |
|  |  |  |  |  |  |  |  |
| Bias in selection of the reported result | Low | Moderate | Moderate | Low | Moderate | Moderate |  |
|  |  |  |  |  |  |  |  |
| Overall bias | Low | Moderate | Moderate | Moderate | Moderate | Moderate |  |
|  |  |  |  |  |  |  |  |
